# Two Decades of Endocrine Silence: Severe Hyponatremia Presenting as Late-Onset Sheehan Syndrome

**DOI:** 10.7759/cureus.113377

**Published:** 2026-07-25

**Authors:** Alberto Jose Sanjuanelo Fontalvo, Said Marien Clarete de la Rosa, Jorge Fernando Quintero Arrieta, Rafael Enrique Camacho Perez, Luis Antonio Rodriguez Arrieta

**Affiliations:** 1 Internal Medicine, University of Cartagena, Cartagena, COL; 2 Internal Medicine, Hospital Universitario del Caribe, Cartagena, COL; 3 Obstetrics and Gynecology, University of Cartagena, Cartagena, COL; 4 Endocrinology, Diabetes, and Metabolism, Instituto del Corazón, Medellín, COL

**Keywords:** empty sella turcica, hypopituiraism, pituitary disorders, postpartum bleeding, sheehan’s syndrome, treatment of hyponatremia

## Abstract

Sheehan syndrome is a rare but potentially fatal complication of postpartum hemorrhage caused by ischemic necrosis of the anterior pituitary gland. Although its incidence has decreased in countries with comprehensive obstetric care systems, it remains a significant cause of panhypopituitarism in regions lacking timely and adequate access to perinatal care. The clinical challenge lies in its insidious presentation: symptoms develop slowly and gradually, and the diagnosis may remain unrecognized for decades.

We report the case of a 52-year-old woman who presented with a one-year history of progressive decline in general health, characterized by debilitating asthenia, somnolence, decreased appetite, and an unintentional 13-kg weight loss. Her obstetric history provided the crucial diagnostic clue: 21 years prior, her last delivery - an initially unattended home birth - was complicated by a retained placenta and severe hemorrhage, followed by total agalactia and permanent secondary amenorrhea. Initial laboratory evaluation revealed severe hyponatremia (119 mmol/L), normocytic anemia, and biochemical evidence of central hypothyroidism and adrenal insufficiency. Magnetic resonance imaging (MRI) confirmed an empty sella turcica. Sequential hormone replacement therapy was initiated - administering glucocorticoids before levothyroxine - resulting in the normalization of serum sodium levels and significant clinical improvement.

The clinical triad of postpartum hemorrhage, agalactia, and secondary amenorrhea constitutes a critical diagnostic red flag. Timely recognition of Sheehan syndrome is essential not only for resolving acute complications such as hyponatremia, driven by secondary hypothyroidism and hypocortisolism, but also for preventing cardiometabolic complications and severe osteoporosis with a high fracture risk associated with untreated chronic hypopituitarism.

## Introduction

Originally described in 1937 by Harold Leeming Sheehan, then Director of Research at the Glasgow Royal Maternity Hospital, this syndrome arises from the physiological hypertrophy of the pituitary gland during pregnancy, rendering it particularly vulnerable to hemodynamic compromise [1,2,3]. When severe postpartum hemorrhage or hypovolemic shock reduces blood flow to this enlarged gland, ischemic necrosis of the anterior lobe can ensue. Sheehan consolidated this pathophysiological understanding through a rigorous anatomopathological study of 11 women who died during the puerperium. Their autopsies provided direct evidence of adenohypophyseal necrosis: nine had a clear history of severe uterine hemorrhage and hypovolemic shock during delivery, whereas the remaining two suffered from severe illnesses before childbirth without massive bleeding. These findings established the causal relationship between obstetric circulatory collapse and pituitary infarction, laying the clinical foundation for the syndrome that now bears his name [1,2].

Advances in obstetric care have significantly reduced its incidence in countries with robust healthcare systems. However, in settings where home births are common, and the management of hemorrhagic complications is delayed or inadequate, Sheehan syndrome remains a leading cause of pituitary insufficiency [3,4].

What makes this disease particularly challenging is its clinical presentation. The initial manifestations - failure to lactate and cessation of menstruation - are often attributed to benign causes or simply dismissed as normal postpartum variations. More profound hormonal deficiencies, such as those affecting the thyroid and adrenal axes, develop so gradually that symptoms are frequently conflated with aging, stress, or common illnesses. The result is a diagnostic delay that, in some cases, can span decades [5].

This prolonged untreated interval is not benign: it exposes patients to potentially lethal complications, including severe hyponatremia, adrenal crises, and progressive cardiovascular deterioration [6].

We present the case of a patient diagnosed with panhypopituitarism 21 years after the inciting obstetric event. The objective of this report is to underscore the importance of maintaining a high index of clinical suspicion when presented with a suggestive obstetric history, regardless of the time elapsed.

## Case presentation

A 52-year-old Hispanic Colombian woman, accompanied by family members, presented to the emergency department in poor general condition, exhibiting acute confusion, an endomorphic body habitus, and puffy facies. Over the past year, she had experienced an insidious decline in her health: fatigue had restricted her daily activities to the point of being nearly bedridden, accompanied by persistent daytime somnolence, decreased appetite, and an unexplained 13-kg weight loss.

Over the preceding five years, she had noted cold intolerance, dry skin, hair loss, chronic constipation, and the gradual disappearance of body hair - symptoms she had assumed were a natural part of aging.

Exploration of her obstetric history revealed the crucial diagnostic clue. In September 2004, her last delivery occurred at home without timely medical assistance. She experienced retained placental fragments, and the ensuing uterine hemorrhage necessitated an emergency transfer to a hospital for manual extraction; despite the activation of a massive obstetric hemorrhage protocol (Code Red), she did not receive a blood transfusion. The patient reported that, despite having successfully breastfed all her previous children, she developed total agalactia, stating clearly that she never produced milk for this infant, and her menses never returned.

On admission, physical examination was notable for generalized pallor of the skin and mucous membranes, as well as a complete absence of axillary and pubic hair. Initial emergency laboratory workup raised immediate concern (Table 1), revealing severe euvolemic hyponatremia (119 mmol/L) and normocytic anemia.

**Table 1:** Initial hematological and biochemical findings.

Laboratory test	Result	Reference range
Hemoglobin	9.4 g/dL	12.0-14.5 g/dL
Hematocrit	25.7%	36.0%-42.0%
Serum sodium	119 mmol/L	136-145 mmol/L
Serum chloride	83 mmol/L	98-107 mmol/L

Given the clinical picture suggestive of myxedema and hyponatremia without an apparent cause, the hormonal profile was expanded. The results confirmed the diagnosis of panhypopituitarism (Table 2): morning cortisol was inappropriately low for the sodium levels, central hypothyroidism was present (inappropriately normal TSH with low free thyroxine levels), alongside marked hypogonadism and nearly undetectable prolactin levels.

**Table 2:** Endocrine profile evaluation. *Ranges adjusted for the expected postmenopausal status based on the patient's age.

Hormonal axis/laboratory test	Result	Reference range	Interpretation
Morning serum cortisol	10.3 µg/dL	5.27-22.45 µg/dL	Inappropriately low (adrenal insufficiency)
TSH	0.93 mUI/L	0.55-4.78 mUI/L	Decreased (central hypothyroidism)
Free thyroxine (FT4)	1.29 µg/dL	4.87-11.72 µg/dL	Decreased (central hypothyroidism)
FSH	1.31 mUI/mL	23.0-116.3 mUI/mL*	Decreased (hypogonadism)
LH	0.1 mUI/mL	15.9-54.0 mUI/mL*	Decreased (hypogonadism)
Prolactin	1.25 ng/mL	4.79-23.3 ng/mL	Decreased (lactotroph deficiency)

**Figure 1:**
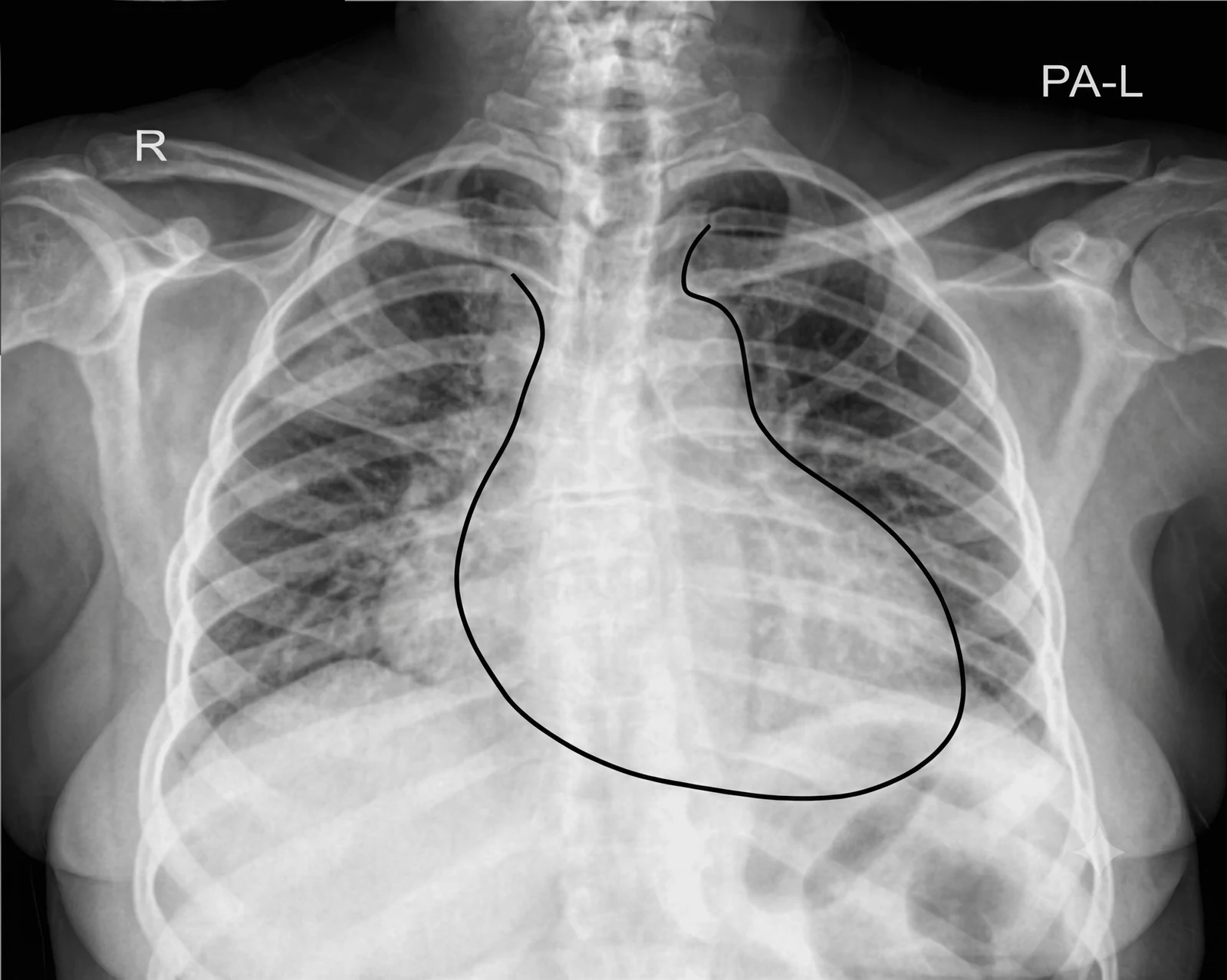
Chest radiograph. Cardiomegaly (enlarged heart outlined in black). No nodular opacities or pulmonary consolidations.

Imaging studies completed the clinical picture. A chest radiograph revealed cardiomegaly; the cardiothoracic ratio (CTR) of this patient was approximately 0.59 (Figure 1), later confirmed by a transthoracic echocardiogram showing a preserved ejection fraction of 57%, a moderate pericardial effusion with a maximum separation of 18 mm, and concentric left ventricular remodeling with a borderline-low ejection fraction.

**Figure 2:**
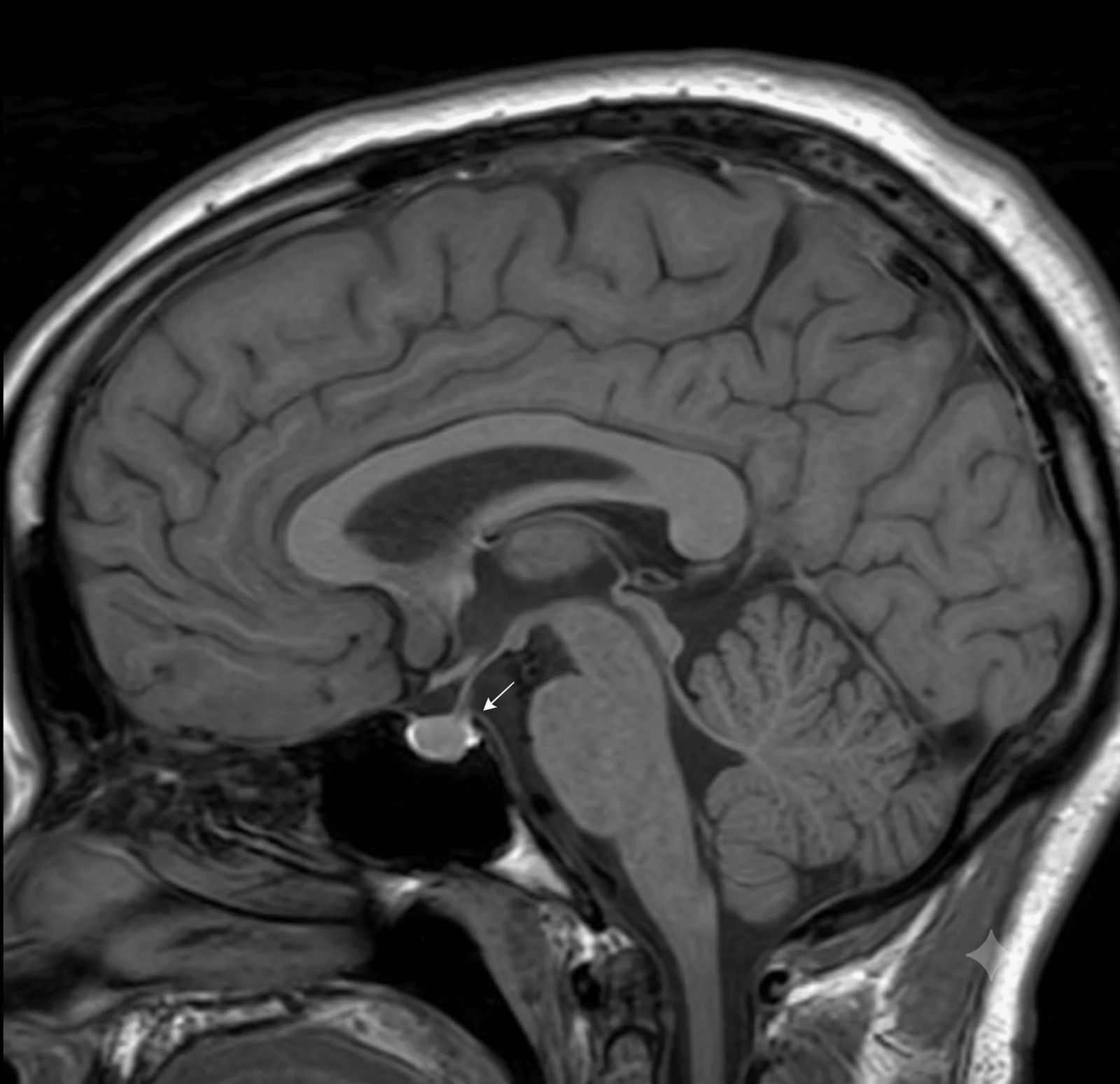
MRI of the sella turcica. Normal-sized sella turcica with an absence of identifiable adenohypophyseal tissue, confirming the diagnosis of empty sella (white arrow).

Magnetic resonance imaging (MRI) of the sellar region was conclusive: a sella turcica of normal dimensions but with a complete absence of identifiable adenohypophyseal tissue, confirming an empty sella (Figure 2).

With the established diagnosis of panhypopituitarism secondary to Sheehan syndrome, hormone replacement therapy was initiated, adhering strictly to the therapeutic sequence of administering glucocorticoids prior to thyroid hormone. Intravenous hydrocortisone was administered first, followed by levothyroxine. The clinical response was remarkable. Over the following days, serum sodium gradually rose to 135 mmol/L, somnolence resolved, and the patient progressively regained her functional capacity. The pericardial effusion improved spontaneously following the initiation of levothyroxine, driven by the positive therapeutic response to thyroid hormone replacement therapy. Consequently, specific drainage interventions were not required, as hormone supplementation alone led to the progressive resolution of the effusion. She was discharged in stable condition on oral levothyroxine (75 mcg daily) and oral prednisolone (5 mg daily), and was scheduled for outpatient endocrinology follow-up.

## Discussion

Time between delivery and diagnosis: a gap measured in decades

This woman's case is, in many respects, representative of what the literature describes regarding Sheehan syndrome: a prolonged period of clinical silence in which the patient lives with symptoms that no one links to the inciting obstetric event. With a 21-year interval to diagnosis, her history aligns perfectly with the average reported in large series, where the mean time to disease identification ranges from 19.7 to 20.4 years [1,2].

This delay is not coincidental. The pathophysiology of the syndrome explains its silent clinical behavior: ischemic necrosis preferentially affects the most vulnerable cells of the anterior lobe, located in areas with less collateral circulation. Lactotroph and gonadotroph cells are the first to succumb, explaining the immediate postpartum agalactia and amenorrhea. Corticotroph and thyrotroph cells, conversely, are more resilient; their failure develops insidiously, presenting with symptoms that medical professionals-and the patients themselves-tend to minimize or attribute to other causes [3,5].

Added to this biological factor is a structural dimension: in women with a small sella turcica volume, the hypertrophied pituitary tissue during pregnancy becomes especially compressed during the hypoperfusion episode. Volumetric studies have demonstrated that patients with Sheehan syndrome have a sellar volume up to 40% smaller than healthy controls (340-350 mm³ vs. 565-602 mm³), which could explain their heightened susceptibility to ischemic damage [1,5,6].

Hyponatremia as the initial presentation: a diagnosis demanding thorough investigation

The severe hyponatremia at admission in this patient - 119 mmol/L - is a well-described manifestation of panhypopituitarism, although it remains underdiagnosed as such [7]. Its mechanism is multifactorial. Cortisol deficiency stimulates the inappropriate release of vasopressin and generates peripheral vasodilation with a reduction in effective arterial volume. Concurrently, central hypothyroidism decreases the glomerular filtration rate and impairs the renal capacity to excrete free water. The result is hyponatremia that can be clinically confused with the syndrome of inappropriate antidiuretic hormone secretion (SIADH) [8].

This similarity is not trivial. When faced with hyponatremia of seemingly central origin, it is imperative to actively rule out adrenal insufficiency and hypothyroidism before labeling it as SIADH, as the wrong treatment can result in an adrenal crisis precipitated by fluid restriction or the use of vasopressor agents. Our patient's case clearly illustrates this diagnostic pitfall and reinforces the need for a comprehensive hormonal profile in any dysnatremia of uncertain etiology.

Triad that should not be forgotten

In retrospect, the most telling signs of this woman's disease were over two decades old: total agalactia since the puerperium and permanent secondary amenorrhea. These two findings, coupled with a history of severe postpartum hemorrhage, constitute the classic triad of Sheehan syndrome and are sufficient, on their own, to guide the diagnosis [3,9].

The literature documents that agalactia occurs in up to 88% of cases and secondary amenorrhea in 96%, reflecting the early and consistent involvement of lactotroph and gonadotroph cells [5]. The fact that these two events did not prompt an endocrinological investigation at the time speaks to a recognition problem that persists in clinical practice, particularly in settings where postpartum follow-up is limited.

Heart as a silent victim of hypopituitarism

The moderate pericardial effusion documented in this patient adds a cardiovascular dimension to the clinical picture. Far from being a rarity, cardiac alterations are frequent in untreated hypopituitarism: pericardial effusion has been reported in up to 48% of patients with Sheehan syndrome, accompanied by mitral regurgitation and reduced left ventricular mass [10]. Fortunately, these alterations are reversible with appropriate hormone replacement therapy, as confirmed by echocardiographic follow-up series [10,11].

Beyond acute manifestations, untreated Sheehan syndrome establishes a profile of accelerated cardiovascular risk. Recent studies have documented coronary calcification in over 50% of patients and atherosclerotic plaques in nearly a quarter of them on coronary computed tomography angiography [12,13]. This risk is explained by the convergence of dyslipidemia, abdominal obesity, insulin resistance, chronic low-grade inflammation, and endothelial dysfunction-all consequences of prolonged hypopituitarism [14].

Added to these cardiovascular complications are non-alcoholic fatty liver disease, present in approximately 63% of patients-with half developing severe steatosis-and bone loss, with osteoporosis in 60% and osteopenia in 30% of cases [5,15]. Every undiagnosed year silently represents cumulative end-organ damage that is difficult to fully reverse.

Appropriate therapeutic sequence as a safety imperative

The management of this patient illustrates a rule that admits no exceptions: in panhypopituitarism, glucocorticoids must always be initiated before thyroid hormones. Levothyroxine accelerates cortisol metabolism; administering it to a patient with untreated adrenal insufficiency can precipitate a potentially fatal adrenal crisis [16].

In practice, hydrocortisone is administered in divided doses, with a larger dose upon awakening to replicate the physiological circadian peak, and a second, smaller dose in the afternoon. The usual daily dose of 20 mg of hydrocortisone is roughly equivalent to 5 mg of prednisone or 0.5 mg of dexamethasone. Follow-up relies on clinical evaluation, as no biomarker reliably reflects the adequacy of glucocorticoid replacement [16].

In premenopausal women with hypogonadotropic hypogonadism, estrogen replacement-preferably via the transdermal route to minimize effects on coagulation and the lipid profile-is essential to prevent the cardiovascular and bone-related consequences of chronic estrogen deprivation [16].

Regarding growth hormone replacement, although not implemented in this case, there is evidence of benefit on the cardiometabolic profile in patients with Sheehan syndrome. The decision must be individualized, considering the cost, adverse effects, and comorbidities of each patient [9].

Active surveillance as a tool for early diagnosis

This case invites us to reconsider the role of the healthcare system in monitoring women with a history of severe postpartum hemorrhage. The implementation of structured postpartum follow-up protocols, including the assessment of lactation, return of menses, and a basic hormonal evaluation, could facilitate the early detection of a significant number of cases before the disease produces established end-organ damage.

Equally important is the continuous medical education of healthcare personnel. Sheehan syndrome is not a disease of the past, nor is it exclusive to low-resource settings. While its prevalence among migrant populations in developed countries is higher than commonly recognized, this case involves a non-immigrant native patient [17], and unfamiliarity with the entity remains one of the primary obstacles to its timely diagnosis [4].

## Conclusions

Sheehan syndrome is not a disease of the past. It is an underdiagnosed entity that can remain hidden for decades, silently accumulating end-organ damage that is not always reversible. This case serves as a paradigm: 21 years elapsed between the hemorrhage that silently destroyed this patient's pituitary gland and the day she presented to the emergency department in a state of acute decompensation.

The fundamental takeaway is simple and enduring: a history of severe postpartum hemorrhage followed by a failure to lactate and permanent cessation of menstruation must trigger an endocrinological evaluation, regardless of how many years have passed. This clinical triad has no expiration date.

When confronted with refractory hyponatremia, unexplained normocytic anemia, serous effusions, or chronic asthenia in a woman with this obstetric history, panhypopituitarism must be at the forefront of the clinician's differential diagnosis. Once confirmed, the therapeutic sequence - glucocorticoids first, followed by levothyroxine - is not merely a recommendation; it is a critical safety measure.

Early diagnosis does more than resolve the immediate crisis. It is, ultimately, the only way to prevent the silent deterioration that untreated hypopituitarism inflicts on the heart, liver, and bones, and to preserve the overall quality of life for those afflicted.
